# Importance of Insulin Resistance in the COVID-19 Era: A Retrospective Analysis of a Single Center in Mexico

**DOI:** 10.7759/cureus.29542

**Published:** 2022-09-24

**Authors:** Ana L Peralta Amaro, Julio C Ramírez Ventura, Luis R Bañuelos García, Emily I Pecero García, José G Valadez Calderón, Rosa N Hernández Flandes

**Affiliations:** 1 Internal Medicine, Hospital de Especialidades Dr. Antonio Fraga Mouret, Instituto Mexicano del Seguro Social, Mexico City, MEX; 2 Endocrinology and Diabetes, Hospital de Especialidades Dr. Antonio Fraga Mouret, Instituto Mexicano del Seguro Social, Mexico City, MEX

**Keywords:** covid-19 retro, mortality, covid-19, glucose, homa-ir, insulin resistance

## Abstract

Introduction and objectives

Type 2 diabetes mellitus (T2DM) has been one of the main risk factors associated with mortality from the coronavirus disease 2019 (COVID-19). Insulin resistance (IR) is a preceding and underlying condition of T2DM, which has been thought that it could increase mortality from COVID-19 since it favors the entry of severe acute respiratory syndrome coronavirus type 2 in the host cell. This article reports a biochemical study that estimated the prevalence of IR in COVID-19 patients and non-diabetic patients without COVID-19 history. It also assesses the prognostic role of IR in the evolution of patients with COVID-19.

Materials and methods

In this single-center, retrospective and cross-sectional design, we included patients with severe and critical COVID-19 and non-diabetic patients without COVID-19 history. We calculated the Homeostatic Model Assessment Insulin Resistance (HOMA-IR) and defined IR with a HOMA-IR >2.6. We estimated the prevalence of IR in both groups and used *x*^2^ to assess the association between IR and mortality from severe and critical COVID-19.

Results

One hundred and twenty-three COVID-19 patients were included with a mean age of 53±15 years: 77 (62.6%) were men and 46 (37.4%) were women. Eighty (65%) patients were critical while the rest were severe. Forty-three (35%) patients died. Seventy-one (57.7%) patients had IR; there was no evidence of an association between IR and mortality from severe or critical COVID-19. Fifty-five non-diabetic patients without COVID-19 history were included with a median age of 40 (26-60) years; 35 (63.6%) were men and 20 (36.4%) were women. Nineteen (34.5%) people had IR.

Conclusion

IR was more prevalent in patients with severe and critical COVID-19 than in non-diabetic patients without COVID-19 history. Our results showed no evidence of the association between IR and mortality from severe and critical COVID-19.

## Introduction

Just over two years after the coronavirus disease 2019 (COVID-19) was declared a pandemic, it has become one of the leading causes of death worldwide with 6,410,961 deaths reported by the World Health Organization [1].

Several studies have highlighted type 2 diabetes mellitus (T2DM) as one of the main risk factors for developing severe COVID-19; this is to say that there is a higher risk of mortality from this disease in diabetic patients compared to non-diabetics [2-7]. The prevalence of T2DM in patients with severe COVID-19 ranges between 22.2% and 26.9% [3]; in Mexico, for example, the prevalence in this group of patients ranges between 30.3% and 39.39% [4,5].

T2DM is characterized by an inflammatory state with endothelial damage that, it is thought, could favor a fatal outcome in patients with COVID-19 [8,9]; however, this state is already present before the clinical and biochemical diagnosis of T2DM in a context known as insulin resistance (IR) [10].

IR favors overexpression of angiotensin-converting enzyme 2 (ACE2) receptors, an enzyme necessary for the entry of severe acute respiratory syndrome coronavirus type 2 (SARS-CoV-2) into the host cell [7,11,12]; as a result, people with IR are expected to have a higher viral load. On the other hand, SARS-CoV-2 can cause direct pancreatic damage in up to 17% of COVID-19 patients [13]. This pancreatic damage could alter insulin secretion and cause the development of IR, which would lead to a vicious circle with greater expression of ACE2 and, consequently, greater entry of the virus into the body.

Although IR is associated with overweight and obesity, it can also be seen in non-obese young people without other obvious metabolic disorders. Similarly, there are non-diabetic patients, without any other comorbidity, who die from COVID-19. This has led us to question whether these patients already had an undiagnosed state of IR that would induce the appearance of a severe case of COVID-19 and, for that reason, a higher risk of mortality from this disease.

Finally, some studies have postulated that COVID-19 infection is a risk factor for the subsequent development of IR [14].

To determine whether IR is a risk factor for mortality, we conducted a biochemical study that evaluated the prognostic role of IR in the evolution of patients with severe and critical COVID-19 admitted to our hospital. Likewise, we estimated the prevalence of IR in non-diabetic patients without a history of COVID-19.

## Materials and methods

Patients

In this cross-sectional design, the main objective was to estimate the prevalence of IR in COVID-19 patients and in non-diabetic patients without COVID-19 history. The secondary objective was to estimate the association between IR and mortality in patients with severe and critical COVID-19.

We included 123 consecutive patients aged 18 years and above, with confirmed COVID-19 diagnosis by reverse transcription-polymerase chain reaction (RT-PCR) assays in nasopharyngeal swab samples, and admitted to the hospitalization area of the Hospital de Especialidades del Centro Médico Nacional La Raza, IMSS in Mexico City, from June 1st to September 30th, 2020. Patients diagnosed with diabetes mellitus, liver disease, hypothalamic-pituitary-gonadal axis disease, human immunodeficiency virus infection; pregnant women, and patients under treatment with steroids before admission were not included. This protocol was approved by the Research Committee and the Research Ethics Committee of the Hospital with registration number R-2020-3501-144. Informed consent was obtained from all participants.

In the group of non-diabetic patients without COVID-19 history, we included 50 participants between January and February 2022. This protocol was approved by the Research Committee and the Research Ethics Committee of the Hospital with registration number R-2021-3501-081. Informed consent was obtained from all participants.

Upon admission, COVID-19 patients were examined to determine the severity of the disease according to the "COVID-19 treatment guidelines" published by the National Institutes of Health: (1) mild: mild symptoms, no pneumonia in imaging diagnosis; (2) moderate: fever, respiratory tract symptoms, and pneumonia in imaging diagnosis; (3) severe: either respiratory rate >30 beats/min, or finger oxygen saturation <93% at rest, or arterial blood oxygen partial pressure/oxygen concentration <300 mm Hg; or (4) critical: respiratory failure requiring mechanical ventilation, organ failure requiring care in the intensive care unit, or shock [15]. A blood sample was taken from each patient by peripheral puncture the first day after their hospital admission between 06:00 am and 08:00 am. The biochemical analysis included hematic biometry, glucose, insulin, creatinine, albumin, and lactate dehydrogenase (LDH). In the group of non-diabetic patients without COVID-19 history, a blood sample was taken after they fasted for 8 hours, and the biochemical analysis included glucose and insulin. We calculated the Homeostatic Model Assessment Insulin Resistance (HOMA-IR) with the following formula: fasting serum glucose (mg/dL) x fasting insulin (mUI/L)/405. We defined IR with a HOMA-IR >2.6 (sensitivity 82% and specificity 65%), which has been standardized for the Mexican-American population [16]. Demographic characteristics, comorbidities, and outcomes were obtained from the medical record.

Statistical analysis

Descriptive statistics were performed on all data. Categorical variables were expressed in number and percentage, and quantitative variables as mean with standard deviation or median with range. The Kolmogorov-Smirnov test was used to know the distribution of the variables. Student's t-test and Mann-Whitney U test were used depending on the variable distribution. To compare categorical variables, we used X2. A p<0.05 value was considered statistically significant. The receiver operating characteristic (ROC) curve was used to obtain the mortality cut-off points of variables of interest such as age, glucose, IR, and LDH, according to the higher sensitivity (Sn%) and specificity (Sp%) shown in the analysis while calculating the positive predictive value (PPV%), and negative predictive value (NPV%). The ROC curve was used to obtain the glucose and insulin cut-off points from which patients with severe and critical COVID-19 developed IR, and from which non-diabetic patients with no history of COVID-19 developed it. The area under the curve (AUC) was reported. We used IBM SPSS statics version 25 (IBM Corp., Armonk, NY).

## Results

Severe and critical COVID-19 patients

One hundred and twenty-three patients were included with a mean age of 53±15 years; 77 (62.6%) were men and 46 (37.4%) were women. Eighty (65%) patients had a critical illness and the rest had a severe illness. Hypertension was the most frequent comorbidity (37.4%), followed by obesity (37.4%), chronic kidney disease (8.9%), cardiovascular diseases (CVD) (6.5%), cancer (4.9%), hypothyroidism (4.1%), dyslipidemia (3.3%), and autoimmune rheumatologic disease (1.6%) (Tables 1, 2).

**Table 1:** Univariate analysis of demographic and biochemical characteristics of surviving and non-surviving patients due to COVID-19 Statistical analysis: Student's t-test and Mann-Whitney U test. Data is expressed as frequency (percentage) for categorical variables; mean ± standard deviation, and median (interquartile range) for continuous parameters. *BMI: Body mass index. †HOMA: Homeostatic Model Assessment for Insulin Resistance. ‡LDH: Lactate dehydrogenase.

	Reference parameters	All patients n = 123 (100%)	Survivors n = 80 (65%)	Non-survivors n = 43 (35%)	p
Age, years	-------	53 ± 15	49 ± 15	60 ± 13	<0.0001
BMI*	-------	28.7 ± 4.7	28.7 ± 5.1	28.7 ± 3.8	0.988
Glucose, mg/dL	70-105	106 (61-342)	100.5 (61-342)	115 (64.6-282)	0.056
Insulin, mUI/mL	0.1-29.1	10.7 (0.2-59.9)	10.95 (1.6-59.9)	10 (0.2-36.9)	0.146
HOMA^†^	-------	2.83 (0.05-18.66)	2.81 (0.32-18.32)	2.84 (0.05-18.66)	0.811
Albumin, g/dL	3.4-5.0	3.5 ± 0.6	3.6 ± 0.5	3.4 ± 0.7	0.118
LDH^‡^, UI/L	123-245	439 (115-17057)	392 (115-1258)	593 (229-17057)	<0.0001
Lymphocytes, 10^9^/L	1.5-4.0	928 (139-5951)	965 (139-4019)	846 (333-5951)	0.592

**Table 2:** Bivariate analysis of demographic and laboratory characteristics of surviving and non-surviving COVID-19 patients Statistical analysis: X2 and ROC curves. Data is expressed as frequency (percentage) for categorical variables. *ROC cut-off points for COVID-19 mortality were obtained according to the higher sensitivity and specificity shown in the analysis. †HOMA-IR: Homeostatic Model Assessment for Insulin Resistance. ‡Lymphopenia: Lymphocytes <1.0 x 10^9^/L. §LDH: Lactate dehydrogenase. ΩHypoalbuminemia: Albumin <3.5 g/dL.

	All patients n = 123 (100%)	Survivors n = 80 (65%)	Non-survivors n = 43 (35%)	p
Age >53 years*	61 (49.6%)	31 (38.8%)	30 (69.8%)	0.001
Male	77 (62.6%)	51 (63.7%)	26 (60.5%)	0.720
Female	46 (37.4%)	29 (36.3%)	17 (39.5%)	
Severe COVID-19	80 (65%)	73 (91.3%)	7 (16.3%)	<0.0001
Critical COVID-19	43 (35%)	7 (8.8%)	36 (83.7%)	
Glucose >107 mg/dL*	59 (48%)	33 (41.3%)	26 (60.5%)	0.042
Insulin <10.3 mUI/mL*	57 (46.3%)	34 (42.5%)	23 (53.5%)	0.244
HOMA-IR^†^ >2.6	71 (57.7%)	45 (56.3%)	26 (60.5%)	0.652
Lymphopenia^‡^	68 (55.3%)	17 (21.3%)	26 (60.1%)	0.450
LDH^§^ >424 IU/L*	66 (53.7%)	35 (43.8%)	31 (72.1%)	0.003
Hypoalbuminemia^Ω^	39 (31.7%)	21 (36.2%)	47.4 (47.4%)	0.276
Hypertension	46 (37.4%)	31 (38.8%)	15 (34.9%)	0.673
Obesity	46 (37.4%)	32 (40%)	14 (32.6%)	0.442
Chronic kidney disease	11 (8.9%)	10 (12.5%)	1 (2.3%)	0.059
Cardiovascular disease	8 (6.5%)	3 (3.8%)	5 (11.6%)	0.091
Cancer	6 (4.9%)	4 (5%)	2 (4.7%)	0.932
Hypothyroidism	5 (4.1%)	2 (2.5%)	3 (7%)	0.231
Dyslipidemia	4 (3.3%)	4 (5%)	0 (0%)	0.136
Autoimmune disease	2 (1.6%)	2 (2.5%)	0 (0%)	0.296

For analysis, the patients were divided into survivors (65%, n=80) and non-survivors (35%, n=43). Non-survivors (NSv) were older and had higher LDH levels than survivors (Sv). There were no differences in body mass index, glucose, insulin, HOMA-IR, creatinine, albumin, or lymphocytes (Table 1).

The cut-off points for mortality of the variables of interest were: age >53 (AUC 0.709, Sn 70%, Sp 61%, PPV 49%, NPV 79%), glucose >107 mg/dL (AUC 0.605, Sn 60%, Sp 59%, PPV 44%, NPV 73%), insulin <10.3 mUI/mL (AUC 0.580, Sn 53%, Sp 57%, PPV 40%, NPV 70%), and LDH >424 UI/L (AUC 0.734, Sn 72%, Sp 56%, PPV 47%, NPV 79%).

Age >53 years, glucose >107 mg/dL, LDH >447 IU/L, and critical COVID-19 were associated with COVID-19 mortality. Seventy-one (57.7%) patients had IR; there was no evidence of an association between IR and mortality from severe or critical COVID-19, and so were gender, comorbidities, insulin <10.3 IU/L, lymphopenia, or hypoalbuminemia (Table 2).

When distinguishing between patients with IR (57.7%, n = 71) and those without IR (42.3%, n = 52), we observed higher glucose and insulin levels in patients with IR than in those without (Table 3). Glucose levels >98.5 mg/dL (AUC 0.852, Sn 83%, Sp 71%, PPV 80%, NPV 76%) and insulin levels >7.4 mUI/mL (AUC 0.950, Sn 94%, Sp 65%, PPV 79%, NPV 89%) were associated with the development of IR in patients with COVID-19 (Table 4). There were no differences in age, BMI, albumin, LDH, or lymphocyte levels between the groups. Neither gender nor the presence of comorbidities was associated with IR in patients with severe and critical COVID-19 (Tables 3, 4).

**Table 3:** Univariate analysis of demographic and biochemical characteristics of COVID-19 patients with and without insulin resistance Statistical analysis: Student's t-test and Mann-Whitney U test. Data is expressed as frequency (percentage) for categorical variables; mean ± standard deviation, and median (interquartile range) for continuous parameters. *IR: insulin resistance. †BMI: Body mass index. ‡LDH: Lactate dehydrogenase.

	Reference parameters	All patients n = 123 (100%)	IR* n = 71 (57.7%)	Non-IR* n = 52 (42.3%)	P
Age, years	-------	53 ± 15	53 ± 15	53 ± 16	0.958
BMI^†^	-------	28.7 ± 4.7	29.2 ± 4.9	28 ± 4.4	0.170
Glucose, mg/dL	70-105	106 (61-342)	122 (72-342)	87.3 (61-164)	<0.0001
Insulin, mUI/mL	0.1-29.1	10.7 (0.2-59.9)	13.8 (5.4-59.9)	6.15 (0.2-11.4)	<0.0001
Albumin, g/dL	3.4-5.0	3.5 ± 0.6	3.6 ± 0.5	3.4 ± 0.8	0.317
LDH^‡^, UI/L	123-245	439 (115-17057)	460 (208-3050)	417 (115-17057)	0.232
Lymphocytes, 10^9^/L*	1.5-4.0	928 (139-5951)	930 (139-3717)	895 (173-5951)	0.927

**Table 4:** Bivariate analysis of demographic and laboratory characteristics of COVID-19 patients with and without insulin resistance Statistical analysis: X2 and ROC curves. Data is expressed as frequency (percentage) for categorical variables. *ROC cut-off points for IR development were obtained according to the higher sensitivity and specificity shown in the analysis. †IR: insulin resistance. ‡Lymphopenia: Lymphocytes <1.0 x 10^9^/L. §Hypoalbuminemia: Albumin <3.5 g/dL.

	All patients n = 123 (100%)	IR^†^ n = 71 (57.7%)	Non-IR^†^ n = 52 (42.3%)	p
Male	77 (62.6%)	44 (62%)	33 (63.5%)	0.866
Female	46 (37.4%)	27 (38%)	19 (36.5%)	
Glucose >99 mg/dL*	74 (60.2%)	59 (83.1%)	15 (28.8%)	<0.0001
Insulin >7.4 mUI/mL*	85 (69.1%)	67 (94.4%)	18 (34.6%)	<0.0001
Lymphopenia^‡^	68 (55.3%)	38 (53.5%)	30 (57.7%)	0.646
Hypoalbuminemia^§^	39 (31.7%)	20 (34.5%)	19 (50%)	0.130
Hypertension	46 (37.4%)	28 (39.4%)	18 (34.6%)	0.585
Obesity	46 (37.4%)	29 (40.8%)	17 (32.7%)	0.356
Chronic kidney disease	11 (8.9%)	4 (5.6%)	7 (13.5%)	0.133
Cardiovascular disease	8 (6.5%)	5 (7%)	3 (5.8%)	0.777
Cancer	6 (4.9%)	3 (4.2%)	3 (5.8%)	0.695
Hypothyroidism	5 (4.1%)	2 (2.8%)	3 (5.8%)	0.413
Dyslipidemia	4 (3.3%)	3 (4.2%)	1 (1.9%)	0.477
Autoimmune disease	2 (1.6%)	0 (0%)	2 (3.8%)	0.096

Non-diabetic patients without COVID-19 history

Fifty-five non-diabetic patients without COVID-19 history were included with a median age of 40 (26-60) years; 35 (63.6%) were men and 20 (36.4%) were women. The recorded comorbidities were hypertension (12.7%), obesity (21.8%), chronic kidney disease (1.8%), cancer (1.8%), hypothyroidism (5.5%), dyslipidemia (7.3%), and autoimmune rheumatologic disease (3.6%). No one had CVD (Table 5).

**Table 5:** Demographic and biochemical characteristics of non-diabetic patients without COVID-19 history Statistical analysis: Mann-Whitney U test, X2 and ROC curves. Data is expressed as frequency (percentage) for categorical variables, and median (interquartile range) for continuous parameters. ‡ROC cut-off points for IR development were obtained according to the higher sensitivity and specificity shown in the analysis. *IR: Insulin resistance, †BMI: Body mass index.

	Reference parameters	All patients n = 55 (100%)	IR* n = 19 (34.5%)	Non-IR* n = 36 (65.5%)	p
Age, years	-------	40 (26-60)	40 (26-57)	40 (27-60)	0.664
Male	-------	35 (63.6%)	14 (73.7%)	21 (58.3%)	0.260
Female	-------	20 (36.4%)	5 (26.3%)	15 (41.7%)	
BMI^†^	-------	25.9 (18.6-62.1)	29 (23.3-62.1)	24.2 (18.6-33.5)	0.001
Glucose, mg/dL	70-105	84.8 (65-100)	92.2 (72.3-99.8)	83.1 (65-100)	0.001
Insulin, mUI/mL	0.1-29.1	8.6 (2.2-42.6)	16.1 (11.3-42.6)	7.2 (2.2-13.1)	<0.0001
Glucose >88 mg/dL^‡^	-------	23 (41.8%)	14 (73.7%)	9 (25.0%)	0.001
Insulin >11.5 mUI/mL^‡^	-------	20 (36.4%)	18 (94.7%)	2 (5.6%)	<0.0001
Hypertension	-------	7 (12.7%)	5 (26.3%)	2 (5.6%)	0.028
Obesity	-------	12 (21.8%)	8 (42.1%)	4 (11.1%)	0.008
Chronic kidney disease	-------	1 (1.8%)	0 (0.0%)	1 (2.8%)	0.463
Cancer	-------	1 (1.8%)	1 (5.3%)	0 (0.0%)	0.165
Hypothyroidism	-------	(5.5%)	1 (5.3%)	2 (5.6%)	0.964
Dyslipidemia	-------	4 (7.3%)	3 (15.8%)	1 (2.8%)	0.077
Autoimmune disease	-------	3 (3.6%)	1 (5.3%)	1 (2.8%)	0.640

Nineteen (34.5%) people had IR. Participants with IR had a higher body mass index, glucose, and insulin levels than those without IR. There were no age differences between the groups (Table 5).

Hypertension (p=.028) and obesity (p=.008) were associated with IR; the rest of the comorbidities and sex were not associated with IR. Glucose levels >88 mg/dL (AUC 0.781, Sn 74%, Sp 75%, PPV 61%, NPV 84%) and insulin levels >11.5 mUI/mL (AUC 0.994, Sn 95%, Sp 94%, PPV 90%, NPV 97%) were the cut-off points that were associated with the development of IR in non-diabetic patients without COVID-19 history (Table 5).

## Discussion

Since the outbreak of COVID-19 two years ago, numerous studies have reported that T2DM, obesity, and CVD are strong independent risk factors for mortality from COVID-19 [2-6].

IR is a preceding and underlying condition of T2DM that alters insulin-signaling pathways, leading to impaired metabolic and cardiovascular homeostasis [6,9,10,17]. The IR prevalence varies from one country to another; in European adults, it is 15.5% [18] while in Thailand, Texas-USA (Mexican-American population) and Venezuela, the reported prevalence is 23.3%, 39.1%, and 46.5% respectively [16,19,20]. The IR prevalence in our group of non-diabetic patients without COVID-19 history was similar to that reported in the Mexican-American population [16].

Under normal conditions, when insulin binds with its receptor, it will induce GLUT-4 expression, allowing glucose uptake in metabolically active organs such as skeletal muscle, liver, and adipose tissue. In addition, activation of the phosphatidylinositol-3 kinase (PI-3-kinase) and mitogen-activated protein kinase (MAPK) pathways by insulin maintains the balance between vasodilation, which is dependent on nitric oxide, and the vasoconstrictor action of insulin, dependent on endothelin-1, thus regulating the prothrombotic and inflammatory state in the body [17,21].

In IR, there is increased pancreatic insulin secretion to maintain normal serum glucose levels because metabolic target tissues (skeletal muscle, liver, and adipose tissue) are less sensitive to the biological actions of insulin due to decreased GLUT-4 expression [7,22]. Hyperinsulinemia disrupts the balance between PI-3 kinase and MAPK, signaling pathways in favor of the latter, thereby leading to the dysfunction of the vascular smooth muscle cell with the consequent development of vasoconstriction, inflammation, and a prothrombotic state that causes the appearance of micro and macrovascular complications in DM2 [17,21,23,24].

In IR, due to alteration in the mammalian target protein of rapamycin signaling pathway, incomplete oxidation of fatty acids, and formation of reactive oxygen species, inflammatory cytokines are produced such as TNF α, IL1, IL6, IL8, leukotriene B4, MCP1, galectin 3. These cytokines are also increased to a greater extent in patients with severe COVID-19 [17,21-26].

On the other hand, it has been observed that hyperinsulinemia can induce pulmonary dysfunction in obese patients since it increases airway reactivity by stimulating preganglionic parasympathetic fibers in the dorsal motor nucleus of the vagus and the nucleus ambiguous [24].

Finally, hyperinsulinemia can increase the viral load of SARS-CoV-2 since insulin raises the expression of ACE2 receptors located in the lung, cardiovascular system, pancreas, intestine, adipocytes, and immune cells, among others [23].

Therefore, we can suggest that the deleterious effects of a preexisting IR (altered blood pressure, vascular dysfunction, inflammation, and thrombosis) are added to the cytokine storm produced by SARS-CoV-2, leading to greater severity in patients with COVID-19, in addition to the harmful effects of insulin on lung function. We thought it is important to diagnose IR in COVID-19 patients since it could identify patients with greater susceptibility to developing a severe case of COVID-19.

Reiterer et al. [27] found that glycemia >170 mg/dL was significantly associated with respiratory distress syndrome, mortality, and the need for mechanical ventilation. They measured the levels of C-peptide, a direct indicator of endogenous insulin production, and postulated that hyperglycemia in patients with critical COVID-19 was due to the development of IR and not to a deficiency of pancreatic beta cells. They further observed that IR occurred independently of steroid use. On the other hand, Ilias et al. [28] found that critical COVID-19 patients had higher glucose levels and higher IR than those who remained in general wards, which is consistent with our results. They propose, unlike Reiterer et al., that hyperglycemia in severe COVID-19 patients is due to both compromised insulin secretion and decreased insulin sensitivity; however, this inference could have been influenced by their inclusion of diabetic patients who already have compromised insulin secretion. Like Reiterer et al., they observed that their results were not influenced by dexamethasone administration.

Unlike Reiterer et al. and Ilias et al., we were able to calculate the HOMA-IR at hospital admission; however, our results do not show evidence of the association between IR and mortality from severe and critical COVID-19. Hyperglycemia did show this association (>107 mg/dL), although with a lower cut-off point than what was reported by Reiterer et al. NSv had lower insulin levels compared to Sv, though without statistical significance; this is consistent with compromised insulin secretion in severe cases of COVID-19 reported by Ilias et al. The decreases in circulating insulin levels could be as a result of the direct damage of SARS-CoV-2 against pancreatic beta cells.

Currently, some studies show that COVID-19 is a risk factor for the subsequent development of IR. Chen et al. [14] followed 64 non-diabetic patients who recovered from mild and moderate COVID-19 but did not receive steroids and found that COVID-19 could be a risk factor for IR development. The authors pointed out that they did not have data on the insulin sensitivity of these patients before SARS-CoV-2 infection, which is also the case in ours, so they could not rule out that IR was an underlying state present in their patients. Similarly, they did not rule out that the IR developed in these patients was secondary to the administration of interferon-alpha.

Our study's strength lies in the fact that it is the only one that has measured the HOMA-IR at hospital admission of patients with severe and critical COVID-19, and that has the bias of dexamethasone effect on glucose levels eliminated since the study was conducted before the recommendation for the use of dexamethasone in patients with COVID-19.

Like in other studies, the main limitation was the number of patients included, in addition to the fact that we could not measure serum C-peptide levels. We did not have previous data on insulin sensitivity in our group of COVID-19 patients, and this may have misled us into including diabetic patients who were not known with the diagnosis. Another disadvantage was that we did not calculate the HOMA-IR at hospital discharge, nor months later; so we could not assess whether the HOMA-IR values improved or increased.

Even with these limitations, we believe that the analysis is useful because there are still patients who have not suffered COVID-19, and among these, there may be people who are not yet vaccinated. Moreover, we estimate that 34.5% to 39.1% of our COVID-19 patients already had an IR status in which SARS-CoV-2 was the trigger for a further inflammatory state and, possibly in some cases, the final trigger for the onset of T2DM, which is well known as an independent risk factor for COVID-19 mortality. We can even argue that the percentage of preexisting IR in our COVID-19 patients could be higher if we consider that the participants without diabetes and a history of COVID-19 were a younger population.

## Conclusions

Our understanding of COVID-19 pathophysiology has improved, but it is still not certain why the evolution of this disease is more severe in some patients. In COVID-19, higher mortality has been observed in diabetic patients, but there are non-diabetic patients, who, without any other comorbidity, die from this disease. So it has been suggested that the existence of IR could be an adverse prognostic factor in this disease. It has also been reported that IR favors a greater expression of ACE2 receptors, which allows a greater entry of the virus into host cells and, consequently causes increased production of cytokines with a deleterious prognosis. Although we did not find evidence of the association between IR and mortality from severe and critical COVID-19, it is important to point out that patients with COVID-19 had a higher prevalence than what was reported in the different populations of study. COVID-19 will not be the last pandemic that humanity could face; today's young population with IR will be the future diabetic population that faces new pandemics with an adverse prognosis if preventive measures are not established to avoid it.
